# Cross-sectional association between red blood cell distribution width and regional cerebral tissue oxygen saturation in preterm infants in the first 14 days after birth

**DOI:** 10.3389/fped.2023.1238762

**Published:** 2023-10-31

**Authors:** Yuju Mu, Hua Wang, Mengting Tian, Yong Hu, Yi Feng, Ruifeng Lu, Qi He, Shouliang Jiang, Jinglan Huang, Surong Duan, Dezhi Mu

**Affiliations:** ^1^ Department of Pediatrics, West China Second University Hospital, Sichuan University, Chengdu, China; ^2^ Key Laboratory of Birth Defects and Related Disease of Women and Children, Ministry of Education, Sichuan University, Chengdu, China; ^3^ Department of Clinical Medicine, BinZhou Medical College, Yantai, China

**Keywords:** red blood cell distribution width, regional cerebral tissue oxygen saturation, cerebral hypoxia, near-infrared spectroscopy, preterm neonates

## Abstract

**Background:**

Hypoxia can threaten the metabolic functions of different systems in immature neonates, particularly the central nervous system. The red blood cell distribution width (RDW) has recently been reported as a prognostic factor in neurologic diseases. Herein, we examined the correlation between RDW and regional cerebral tissue oxygen saturation (rcSO_2_).

**Methods:**

This cross-sectional study included 110 preterm infants born at a gestational age (GA) of <32 weeks, or with a birth weight (BW) of <1,500** **g at our institution between January and June 2,022. The rcSO_2_ was monitored using near-infrared spectroscopy, and RDW was extracted from the complete blood count during the first 14 days after birth. RDW and rcSO_2_ measurements were analyzed using a cross-sectional research method.

**Results:**

We divided the study population into two groups, with a mean rcSO_2_ value over the first 14 days. Fifty-three preterm had rcSO_2 _≥_ _55% and 57%_ _<_ _55%. The 14-days-mean in the study population showing an association of lower rcSO_2_ values with higher RDW values. Significantly higher RDW values were observed in the low rcSO_2_ group compared with those in the high rcSO_2_ group. Threshold effect analysis showed that rcSO_2_ decreased with RDW values ≥18% (β, −0.03; 95% CI, −0.04 and −0.02; *p*_ _≥_ _0.0001). After adjusting for potential confounders, an RDW of ≥18% was determined as the predictive cutoff value for preterm infants with low rcSO_2_ (Model I: OR, 3.31; 95% CI, 1.36–8.06; *p*_ _=_ _0.009; and Model II: OR, 3.31; 95% CI, 1.28–8.53; *p*_ _=_ _0.013).

**Conclusions:**

An RDW of ≥18% in the first 14 days is associated with rcSO_2_ of <55% in preterm infants.

## 1. Introduction

Despite recent improvements in perinatal care, brain vulnerability in critically ill preterm newborns still represents a challenge. Brain hypoperfusion during the immediate postnatal period has been reported to be related to cerebral damage in sick preterm infants (1). If not corrected in time, hypoxia can lead to poor clinical outcomes, including cerebral palsy, different brain disabilities or death (2, 3).

### 1.1. Red blood cell distribution width

Red cell distribution width (RDW) is an automatically measured complete blood count (CBC) parameter that reflects the degree of heterogeneity in erythrocyte size (4). The RDW is calculated from the distribution of red blood cell (RBC) volumes, measured by impedance, using the following formula:

$$\begin{array}{r} \mathrm{RDW}\;=\;\mathrm{standard}\;\mathrm{deviation}\;\mathrm{of}\;\mathrm{RBC}\;\mathrm{volume}/\\ \mathrm{mean}\;\mathrm{corpuscular}\;\mathrm{volume}\;(\mathrm{MCV})\;\times \;100 \end{array}$$

The “width” in RDW refers to the width of the volume distribution histogram, expressed as a coefficient of variation. In samples with mixtures of small and large RBCs, the histogram is wider; thus, the RDW value is higher. In contrast, in samples with a homogeneous red cell size, the histogram is narrower; thus, the RDW is lower. The normal reference range for RDW among healthy adults used in most laboratories is 11% (lower reference interval limit) to 15% (upper reference interval limit) (5). In 1987, Dr. Robert Novak reported that the normal range of RDW in infants ranged from 11.5% to 14.5% (6). A recent study of 144 infants reported that the lower reference limit for RDW in premature infants at <72 h of age is 14.2%, while the upper reference limit is 21.8% (4). However, due both to the difference in methods of measuring RBC size, instruments, researchers, laboratory standards, and statistical approaches between laboratories, as well as the ethical and practical challenges unique to pediatric care, thus far, no universal reference range has yet been established for pediatric patients (7).

Recently, RDW levels have been shown to be associated with neonatal mortality and/or significant morbidity, including neonatal sepsis, intrauterine growth restriction (IUGR), and bronchopulmonary dysplasia (BPD), etc., (8–10). The RDW has also been indicated as a useful tool for evaluating the medical conditions of newborns, especially preterm infants (11, 12).

### 1.2. Preterm cerebral oxygen

Regional cerebral tissue oxygen saturation (rcSO_2_) can be measured by using near-infrared spectroscopy (NIRS), a frequently used noninvasive technique (13). RcSO_2_ primarily reflects the brain venous compartment, as it is larger than other compartments; however, it is also sensitive to changes in arterial parameters. RcSO_2_ increases with time after birth, and tends to reach a plateau at 8–10 min after birth (14, 15). However, great individual variability can be seen among patients, and depending on the location. A prior publication provided an rcSO_2_ range in preterm infants of 55%–85% (16). An rScO_2_ value of 55% is close to or below the thresholds reported to be associated with neuronal damage and adverse neurodevelopmental outcome (17).

Conventional pulse oximeter measures arterial oxygen saturation around the limb, meaning that pulse oxygen saturation (SpO_2_) does not include information on local tissue oxygen utilization (18). Cerebral oxygenation is influenced by three main components: the blood oxygen content, cerebral perfusion, and oxygen consumption. Each of these factors are themselves influenced by several other variables, including SpO_2_, blood glucose level, partial pressure of carbon dioxide, blood pressure, and hemoglobin level (19). NIRS measures composite arterial, capillary, and venous blood saturation in the brain tissue; as such, rcSO_2_ can reflect real-time subtle changes in tissue oxygenation in the brain (20). Studies have found that the neonates with adverse outcome (including intraventricular haemorrhage) had significantly lower rcSO_2_ values during the immediate transition, although there was no difference concerning SpO_2_ (21, 22). Therefore, potential cerebral hypoxia could not be identified with SpO_2_ monitoring. Although in a research involving extremely preterm infants, treatment guided by cerebral oximetry monitoring in the first 72 h after birth did not lead to a lower incidence of death or severe brain injury at 36 weeks' postmenstrual age than usual care (23), a previously conducted interventional trial performed by the SafeBoosCII study group demonstrated that the combination of cerebral oxygenation monitoring by NIRS combined with an evidence-based treatment guideline significantly reduced the burden of cerebral hypoxia (24, 25). Other studies have reported high sensitivity of NIRS-detected variables of rcSO_2_ to hypoxemic and ischemic events occurring in preterm infants (26).

### 1.3. Relationship between RDW and rcSO_2_

Due to their ability to deform and flow in the microvascular network, RBCs have many vital physiological functions in our body, including carrying oxygen and carbon dioxide and gas exchange between blood and tissues (27). However, Patel et al. found that when the RDW exceeds 14% in microvascular disorders, the deformability of RBCs decreases (28), which may affect these aforementioned functions. In newborns, ineffective erythropoiesis due to prematurity (4), hemolysis, erythrocyte transfusions, and alterations in osmolality under some pathophysiological conditions can all decrease the ability of RBCs to deform, triggering an increase in RDW, thereby resulting in low microvascular perfusion (29, 30). We hypothesized that a high RDW value would be associated with an increased risk of low rcSO_2_ based on analyses of existing clinical data.

This study was therefore conducted to investigate the relationship between RDW and rcSO_2_ in preterm neonates.

## 2. Methods

### 2.1. Study population

The study population consisted of 110 preterm infants born at <32 weeks or with birth weight (BW) <1,500 g between January and June 2022 at the neonatal intensive care unit (NICU) of the West China Second University Hospital, Sichuan University. The exclusion criteria were as follows: advanced resuscitation in the delivery room, cardiovascular instability requiring treatment with inotropes, anemia, and major congenital or chromosomal anomalies. This study was approved by the Ethics Committee of the West China Second University Hospital, Sichuan University (2022–67). Written and verbal informed consent was obtained from the infants' parents, and the study was registered on ChineseClinicalTrials.gov (09/04/2022, ChiCTR2200058482).

### 2.2. Study design

This study had a cross-sectional design. An EGOS-600B Cerebral/Somatic Oximeter device was used for rcSO_2_ monitoring during the study period, with neonatal NIRS sensors (NIRSensor, Enginmed, A1Z2104110037) placed on the frontoparietal regions of the infants. NIRS monitoring was performed thrice daily for the first 14 days after birth. All data were obtained when the infants were mostly quiet or sleeping in the supine position. No handling or intervention was performed during NIRS monitoring. Recorded values were stored and used for offline analyses. NIRS data analysis was performed after the data were transferred to a computer in Excel format using a customized programmer for the EGOS device. Those with markers likely related to an artifact (during handling, positioning, etc.) were excluded for data cleaning and were averaged afterward.

Blood samples were extracted from all preterm infants once a week on the first and second week of life. The volume for blood sampling was 0.50 ml. RDW values were extracted from the CBC, and the average was calculated. Neonatal and perinatal characteristics, including gestational age (GA), BW, sex, mode of delivery, IUGR, hypertension during pregnancy, chorioamnionitis (CA), gestational diabetes mellitus (GDM), premature rupture of membranes (PROM), multiple pregnancies, overall dose of antenatal corticosteroid treatment, invasive mechanical ventilation, breast feeding, and blood transfusion, were recorded prospectively. Neonatal morbidities included early-onset sepsis (EOS), late-onset sepsis (LOS), and intraventricular hemorrhage (IVH).

### 2.3. Statistical analysis

According to the existing literature and clinical judgment, we divided the study population into two groups, with a mean rcSO_2_ value over the first 14 days of 55% as the cut-off limit. For descriptive analyses, continuous variables are described as the means_ _±_ _standard deviations (SD), and categorical data are presented as numbers and percentages (31). The distribution of covariates in the rcSO_2 _≥_ _55% and rcSO_2 _<_ _55% groups was compared using the *t*-test (normal distribution) or Kruskal–Wallis rank sum test (non-normal distribution) for continuous variables, and the chi-squared test for categorical data (Table 1).

**Table 1:** Baseline characteristics of the study participants (*n*_ _=_ _110).

	rcSO_2 _≥_ _55% (*n*_ _=_ _53)	rcSO_2 _<_ _55% (*n*_ _=_ _57)	Standardized difference	*p*-value
GA, weeks (mean_ _±_ _SD)	31_ _±_ _2	31_ _±_ _2	0.21 (−0.16, 0.59)	0.273
BW, g (mean_ _±_ _SD)	1,524_ _±_ _384	1,432_ _±_ _463	0.21 (−0.16, 0.59)	0.265
RDW, % (mean_ _±_ _SD)	16_ _±_ _1	17_ _±_ _2	0.47 (0.09, 0.84)	0.017
Female (*n*, %)	25 (47%)	23 (40%)	0.14 (−0.24, 0.51)	0.471
VD (*n*, %)	14 (26%)	20 (35%)	0.19 (−0.19, 0.56)	0.325
CA (*n*, %)	23 (43%)	26 (46%)	0.04 (−0.33, 0.42)	0.815
Hypertension during pregnancy (*n*, %)	9 (17%)	11 (19%)	0.06 (−0.31, 0.43)	0.753
GDM (*n*, %)	15 (28%)	19 (33%)	0.11 (−0.27, 0.48)	0.568
IUGR (*n*, %)	6 (11%)	6 (11%)	0.03 (−0.35, 0.40)	0.894
PROM (*n*, %)	19 (36%)	21 (37%)	0.02 (−0.35, 0.39)	0.914
Multiple pregnancies (*n*, %)	21 (40%)	26 (46%)	0.12 (−0.25, 0.50)	0.526
Overall dose of antenatal corticosteroid treatment (*n*, %)	39 (75%)	41 (72%)	0.07 (−0.31, 0.45)	0.717
Invasive mechanical ventilation (*n*, %)	22 (42%)	25 (44%)	0.05 (−0.33, 0.42)	0.803
Breast feeding (*n*, %)	19 (36%)	18 (32%)	0.09 (−0.28, 0.46)	0.636
Blood transfusion (*n*, %)	13 (25%)	23 (40%)	0.34 (−0.03, 0.72)	0.077
IVH (*n*, %)	7 (13%)	11 (19%)	0.17 (−0.21, 0.54)	0.388
EOS (*n*, %)	4 (8%)	8 (14%)	0.21 (−0.16, 0.59)	0.275
LOS (n, %)	4 (8%)	6 (11%)	0.10 (−0.27, 0.48)	0.587

RDW, red blood cell distribution width; rcSO_2_, regional cerebral tissue oxygen saturation; GA, gestational age; SD, standard deviation; BW, birth weight; VD, vaginal delivery; CA, chorioamnionitis; GDM, gestational diabetes mellitus; IUGR, intrauterine growth restriction; PROM, premature rupture of membrane; EOS, early-onset sepsis; LOS, late-onset sepsis; IVH, intraventricular hemorrhage.

**Figure 1:**
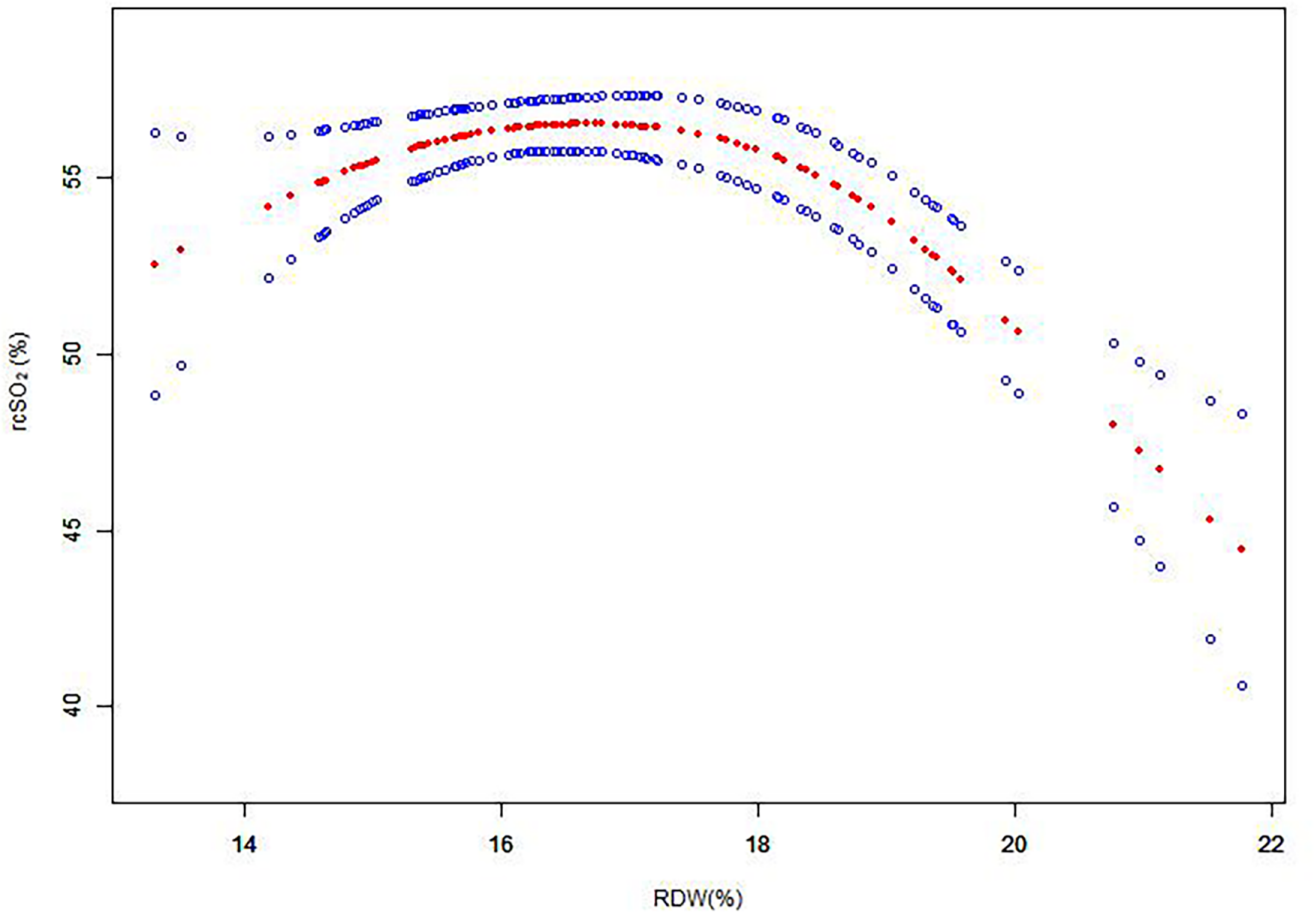
Association between RDW and rcSO_2_ over the first 14 days after birth. A threshold, nonlinear association between RDW and rcSO_2_ was determined (*p*_ _<_ _0.05) from a generalized additive model. Red line: smooth curve fit between variables; blue bands: 95% CI from the fit. RDW, red blood cell distribution width; rcSO_2_, regional cerebral tissue oxygen saturation; CI, confidence interval.

We used a generalized additive model (GAM) to investigate the dose–response relationship between RDW values and rcSO_2_ (Figure 1), and subsequently used a two-piece-wise linear regression model to examine the threshold effect of the RDW value on rcSO_2_ (Table 2). The threshold level (i.e., inflection point) was determined using a trial-and-error approach. This involved selecting inflection points along a predefined interval, and choosing the inflection point that resulted in the maximum model likelihood. We further conducted a log-likelihood ratio test to compare the one-line linear regression model with the two-piecewise linear model, as previously described (32).

**Table 2:** Threshold effect analysis of the RDW and rcSO_2_ over the first 14 days after birth using piece-wise linear regression.

	Effect size (β)	95% CI	*p*-value
Inflection point of RDW			
<18%	0.01	0.00, 0.02	0.038
≥18%	−0.03	−0.04, −0.02	<0.0001
*P* value for LRT test			<0.001

Adjusted for gestational age and birth weight.

RDW, red blood cell distribution width; rcSO_2_, regional cerebral tissue oxygen saturation; LRT, log-likelihood ratio test, CI, confidence interval.

Next, univariate logistic regression (Table 3) and multivariate logistic regression models (Table 4) were applied to examine whether a high RDW was associated with low rcSO_2_ levels in preterm neonates. The statistical results are displayed as odds ratio (OR), with the corresponding 95% CI. We adjusted for variables deemed to be confounders according to the existing literature and clinical judgment. All statistical analyses were performed using EmpowerStats (www.empowerstats.com, X&Y solutions, Inc., Boston, MA) and R version 4.2.0 (http://www.r-project.org).

**Table 3:** Univariate analysis for rcSO_2 _<_ _55% over the first 14 days after birth.

	Statistics	OR (95% CI)	*p-*value
RDW, % (mean_ _±_ _SD)	17_ _±_ _2	1.31 (1.04, 1.65)	0.020
RDW threshold value group			
<18%	78 (71%)	1.00	
≥18%	32 (29%)	3.31 (1.36, 8.06)	0.009
GA, weeks (mean_ _±_ _SD)	31_ _±_ _2	0.91 (0.76, 1.08)	0.272
BW, g (mean_ _±_ _SD)	1478_ _±_ _423	1.00 (1.00–1.00)	0.264
VD (*n*, %)			
No	76 (69%)	1.00	
Yes	34 (31%)	1.51 (0.66, 3.41)	0.327
CA (*n*, %)			
No	61 (55%)	1.00	
Yes	49 (45%)	1.09 (0.52, 2.32)	0.815
Hypertension during pregnancy (*n*, %)			
No	90 (82%)	1.00	
Yes	20 (18%)	1.17 (0.44, 3.09)	0.753
GDM (*n*, %)			
No	76 (69%)	1.00	
Yes	34 (31%)	1.27 (0.56, 2.86)	0.569
IUGR (*n*, %)			
No	98 (89%)	1.00	
Yes	12 (11%)	0.92 (0.28, 3.06)	0.894
PROM (*n*, %)			
No	70 (64%)	1.00	
Yes	40 (36%)	1.04 (0.48, 2.27)	0.914
Multiple pregnancies (*n*, %)			
No	63 (57%)	1.00	
Yes	47 (43%)	1.28 (0.60, 2.73)	0.526
Overall dose of antenatal corticosteroid treatment (*n*, %)			
No	29 (26%)	1.00	
Yes	81 (74%)	0.85 (0.36, 2.00)	0.717
Invasive mechanical ventilation (*n*, %)			
No	63 (57%)	1.00	
Yes	47 (43%)	0.91 (0.43, 1.94)	0.803
Breast feeding (*n*, %)			
No	73 (66%)	1.00	
Yes	37 (34%)	1.21 (0.55, 2.67)	0.636
Blood transfusion (*n*, %)			
No	74 (67%)	1.00	
Yes	36 (33%)	0.48 (0.21, 1.09)	0.080

RDW, red blood cell distribution width; rcSO_2_, regional cerebral tissue oxygen saturation; GA, gestational age; SD, standard deviation; BW, birth weight; VD, vaginal delivery; CA, chorioamnionitis; GDM, gestational diabetes mellitus; IUGR, intrauterine growth restriction; PROM, premature rupture of membrane; CI, confidence interval; OR, odds ratio.

**Table 4:** Relationship between the RDW and rcSO_2_ < 55% over the first 14 days after birth in different models.

Variable	Crude model^a^		Adjusted model^b^	
	OR (95% CI)	*p*-value	OR (95% CI)	*p*-value
RDW, %	1.31 (1.04, 1.65)	0.020	1.31 (1.02, 1.67)	0.036
RDW value group				
<18%	1.00		1.00	
≥18%	3.31 (1.36, 8.06)	0.009	3.31 (1.28, 8.53)	0.013

RDW, red blood cell distribution width; rcSO_2_, regional cerebral tissue oxygen saturation; OR, odds ratio, CI, confidence interval.

^a^ Crude model not adjusted.

^b^ Adjusted model adjusted for gestational age and birth weight.

## 3. Results

### 3.1. Baseline characteristics

This study enrolled 110 preterm neonates, of whom 53 and 57 had rcSO_2_ levels of ≥55% and <55%, respectively. The general characteristics of the study population are summarized in Table 1. There was no noticeable difference between the two groups in terms of GA (31 ± 2 vs. 31 ± 2 weeks; *p *= 0.273), BW (1524 ± 384 vs. 1432 ± 463 g; *p *= 0.265), female (47% vs. 40%; *p *= 0.471), VD (26% vs. 35%; *p *= 0.325), CA (43% vs. 46%; *p *= 0.815), hypertension during pregnancy (17% vs. 19%; *p *= 0.753), GDM (28% vs. 33%; *p *= 0.568), IUGR (11% vs. 10%; *p *= 0.894), PROM (36% vs. 37%; *p *= 0.914), multiple pregnancies (40% vs. 46%; *p *= 0.526), overall dose of antenatal corticosteroid treatment (75% vs. 72%; *p *= 0.717), invasive mechanical ventilation (42% vs. 44%; *p *= 0.803), breast feeding (36% vs. 32%; *p *= 0.636), blood transfusion (25% vs. 40%; *p *= 0.077), IVH (13% vs. 19%; *p *= 0.388), EOS (8% vs. 14%; *p *= 0.275), or LOS (8% vs. 11%; *p *= 0.587).

### 3.2. Association of RDW levels with rcSO_2_

To investigate the association between RDW values and rcSO_2_, the participants were divided into two groups according to the RDW value. The piece-wise linear regression threshold effect analysis of RDW and rcSO_2_ showed that the rcSO_2_ decreased with RDW values >18% (β, −0.03; 95% CI, −0.04– −0.02; *p *< 0.0001) (Figure 1, Table 2). Univariate analysis (Table 3) revealed that the RDW was significantly correlated with preterm rcSO_2_ values <55% over the first 14 days (OR, 1.31; 95% CI, 1.04–1.65; *p *= 0.020). After multivariable risk adjustment for potential confounding factors (Table 4), including GA and BW in the adjusted model, a high RDW was still positively associated with low rcSO_2_ in preterm neonates. In addition, an RDW of ≥18% over the first 14 days could significantly increase the risk of preterm rcSO_2_ values <55% over the first 14 days (adjusted model: OR, 3.31; 95% CI, 1.28–8.53, *p *= 0.013).

## 4. Discussion

Despite significant improvements in survival rates in preterm infants in recent years, the rate of major neurodevelopmental impairment in survivors has not decreased (33). As such, the burden of neurodevelopmental disability due to preterm birth remains a significant challenge for neonatologists and a significant burden on public health. As a result, strategies designed to reduce adverse neurological outcomes have been a major focus of perinatal research.

Experiments on piglets have demonstrated that cerebral oxygen saturation (ScO_2_) values under a certain threshold for prolonged periods are associated with cerebral damage (34). Low rcSO_2_ values were further associated with lower developmental scores in very preterm infants at 2 years of age, and children surviving with sequelae had lower rcSO_2_ values than children without sequelae (35). Further, a direct correlation was found between the time spent with an ScO_2_ of ≤40% and adverse cerebral outcomes or early death (34).

The relationship between SpO_2_ and rcSO_2_ has not been well established. SpO_2_ is not a reliable tool to fully inform about rcSO_2_ (36, 37); therefore, automated FiO_2_ control may be difficult to further improve the stability of rcSO_2_ (38). In adults, current studies have validated that RDW statistically correlates with the National Institutes of Health stroke scale scores and grading in patients with stroke (39). In another study, Söderholm et al. postulated that the incidence of stroke and cerebral infarction increases with RDW level in the general population. In addition, they found evidence of an association between high RDW values and increased intimal-medial thickness in the common carotid artery, which has also been recognized as a risk factor in ischemic stroke (40, 41).

Various factors, including chronic fetal hypoxia *in utero*, infection, inflammation, physiological immaturity of blood cell production, hemolytic anemia, and erythrocyte transfusions, could lead to an elevated RDW in preterm neonates (4, 8, 9, 30). An increased RDW mirrors a profound deregulation of erythrocyte homeostasis (42). Whether RDW plays an active role in health and disease, or simply acts as a biomarker is of increasingly interest and its clinical use should now be broadened beyond its conventional application for troubleshooting anemia.

A retrospective study of 596 critically ill pediatric patients by Ramby et al. found an association between RDW and prolonged pediatric intensive care unit (PICU) stay in those without sepsis, with a 1.17 increased odds for each 1% increase in RDW (6). An elevated RDW in preterm newborns and infants with IUGR was also significantly associated with early mortality (6), as well as worse clinical parameters, including PICU mortality. Thus, RDW could be a promising prognostic factor with the advantages of simple and easy measurement in critically ill pediatric patients (43).

Our data suggest that RDW over the first 14 days could be a useful indicator. The influence of some confounding factors, such as GA, BW, and nursing factors, were excluded by multivariate logistic regression models. Our study identified a statistically significant positive correlation between the RDW and preterm rcSO_2_. An RDW of ≥18% over the first 14 days could significantly increase the risk of a preterm rcSO_2_ of <55%. This value may be applied in clinical practice and could be used during routine monitoring processes for preterm rcSO_2_ in the NICU.

Several high-risk factors that may increase RDW should be avoided in the early stages to reduce cerebral hypoxia injury in preterm infants. For example, identifying the strict indications of erythrocyte transfusions, infection prevention and control, and avoiding fetal hypoxia *in utero* are all important factors. Overall, our findings emphasize the necessity of improving medical measures to decrease the incidence of cerebral hypoxia in preterm neonates.

This study has several limitations. First, the sample size was small, and all patients were enrolled from a single center, which may have restricted the accuracy and generalizability of the results. Moreover, we did not collect SpO_2_ values during the measurement period, and the exact relationship between SpO_2_ and rcSO_2_ therefore could not be elucidated. Further research is needed to explore this factor.

## 5. Conclusion

Overall, the results of the present study showed that an RDW of ≥18% in the first 14 days is associated with rcSO_2_ of <55% in preterm infants. RDW is an easy, simple, inexpensive, and rapid tool that could be used to ensure the early prediction of preterm infants with low rcSO_2_, which is correlated with cerebral hypoxia.

## Data Availability

The raw data supporting the conclusions of this article will be made available by the authors, without undue reservation.
